# In ovo versus ex ovo incubation differentially shapes chorioallantoic membrane maturation, angiogenesis, and tumor growth

**DOI:** 10.1038/s41598-026-49692-9

**Published:** 2026-04-25

**Authors:** Zuzana Demcisakova, Shari Aerts, Julia Hurnikova, Lenka Luptakova, Lukas Urban, Matus Coma, Peter Gal, Liesbeth Couck, Ward De Spiegelaere, Eva Petrovova

**Affiliations:** 1 Department of Morphological Disciplines, University of Veterinary Medicine and Pharmacy in Kosice, Kosice, Slovakia; 2 Department of Morphology, Imaging, Orthopedics, Rehabilitation and Nutrition (DI11), Ghent University, Ghent, Belgium; 3 CRIG (Cancer Research Institute Ghent), ConSarGhent, Ghent University, Ghent, Belgium; 4 VON (Veterinary Oncology Network) - CRIG (Cancer Research Institute Ghent), Ghent University, Ghent, Belgium; 5 Department of Biology and Physiology, University of Veterinary Medicine and Pharmacy in Kosice, Kosice, Slovakia; 6 Department of Pharmacology, Faculty of Medicine, Pavol Jozef Safarik University in Kosice, Kosice, Slovakia; 7 Department of Biomedical Research, East-Slovak Institute of Cardiovascular Diseases Inc, Kosice, Slovakia; 8 Technology and Innovation Park, Pavol Jozef Safarik University in Kosice, Kosice, Slovakia; 9 Prague Burn Centre, Third Faculty of Medicine, Charles University, University Hospital Kralovske Vinohrady, Prague, Czech Republic

**Keywords:** Allantois, Chicken embryo, Chorioallantoic membrane, Chorion, Oncology, Tumor research, Cancer, Cell biology, Developmental biology

## Abstract

The chorioallantoic membrane (CAM) is a highly vascularized avian extraembryonic membrane, widely used as an in vivo model for angiogenesis, with predominant application in tumor research. CAM assays are performed under in ovo or ex ovo conditions; however, a clear rationale for model selection is lacking, and comparative morphological data remain limited. In this study, we compared chicken embryo CAM development under both incubation conditions. Morphological analyses revealed pronounced incubation-dependent differences in CAM maturation, with the mesoderm showing the most dynamic alterations. Ex ovo CAMs exhibited early mesodermal thickening, and peaks in angiogenesis; indicative of accelerated but less coordinated tissue and vascular development. In ovo CAMs showed a gradual and well-coordinated increase in mesodermal thickness and vascularization, peaking at later developmental stages. Differences were also observed in epithelial organization: ex ovo CAMs exhibited disrupted ectodermal architecture, whereas in ovo CAMs maintained stable epithelial stratification. Functional assessment using GFP-labelled human fibrosarcoma cells (HT1080) demonstrated significantly higher embryo survival and larger tumor growth in the in ovo model, while ex ovo conditions were associated with increased mortality and reduced tumor size. These findings demonstrate that incubation conditions significantly influence CAM morphology and functional performance, highlighting the importance of careful model selection in angiogenesis research.

**Supplementary Information:**

The online version contains supplementary material available at 10.1038/s41598-026-49692-9.

## Introduction

Experimental models that accurately reflect angiogenesis, tissue maturation, and tumor-host interactions are central to translational cancer biology, biomaterials research, and regenerative medicine. Small-animal models provide physiological relevance but are associated with high costs, ethical constraints, and limited throughput, prompting growing use of alternative in vivo systems that combine biological complexity with experimental accessibility^1–7^. The chicken chorioallantoic membrane (CAM) assay has emerged as one of the most widely used alternative in vivo platforms for studying angiogenesis, tumor growth, drug responses, tissue and biomaterial integration^3,8,9^. The CAM is a highly vascularized membrane, formed by the fusion of the chorion and the allantois, that serves as the primary respiratory organ of the embryo^10^.

The CAM assay is typically performed using two fundamentally different experimental configurations: the in ovo model, where the embryo develops within the eggshell with a small access window, and the ex ovo model, where the embryo is transferred outside the eggshell early in development^11^. The choice between these models is typically driven by technical convenience rather than biological considerations^12^. For example, the in ovo model is frequently favored in tumor explant studies, while the ex ovo model is commonly selected for angiogenesis observation due to unrestricted access to the vascular network and simplified experimental manipulation^8,12–14^.

Despite the widespread use of the CAM assay, the biological equivalence of in ovo and ex ovo approaches has not been systematically evaluated^12^. Existing comparisons largely focus on survival or practical feasibility, reporting significantly higher overall mortality in ex ovo systems, particularly in the first few days post-transfer, likely due to the initial stress of shell removal^15,16^. Furthermore, most ex ovo grown embryos cannot survive past embryonic day (ED) 18 due to insufficient calcium and phosphate, which are normally absorbed from the eggshell^17,18^. Crucially, no comprehensive data are available on potential differences in the morphology or ultrastructure of the CAM tissue incubated in the in ovo and ex ovo conditions. Such morphological variations could fundamentally alter tissue responses and impact experimental procedures and outcomes.

Understanding these baseline differences is relevant given the tightly regulated developmental timeline of the CAM. The CAM is formed between ED4 and ED5 through the fusion of the chorion and the allantois, resulting in three distinct layers: the outer ectoderm (from the chorion), the central mesoderm, and the inner endoderm (from the allantoic epithelium)^6,10^. The mesoderm, which contains the extensive vascular network, is formed by the fusion of the somatic mesoderm (from the chorion) and the splanchnic mesoderm (from the allantois)^6,19^. The CAM’s continuous vessel system first appears as an immature network around ED4, mainly consisting of blood vessels, which connect with the embryonic circulation^4,20^. This vascular network is highly angiogenic, undergoing maturation by constant generations of new vessels until approximately ED11^21^. The capillary plexus continues to expand, attaining its final, mature arrangement between ED12 and ED14^4,6,20^. This high vascularity of the CAM is a major advantage for research, making it ideal for real-time angiogenesis studies, drug delivery and toxicity assessments, and wound healing models^3,4,20,22–24^. Additionally, the CAM model is naturally immunodeficient until approximately ED15, which enables efficient xenograft engraftment by minimizing early immune-mediated rejection^6,25,26^.

This study addresses the existing knowledge gap by performing a direct comparative analysis of the in ovo and ex ovo CAM assays. Using a combination of histological, ultrastructural, molecular, and tumor xenograft analyses, we identify incubation-dependent differences that impact angiogenesis, embryo survival, and tumor growth, providing a biological framework to guide rational selection of CAM models for experimental and translational research.

## Results

**Fig. 1:**
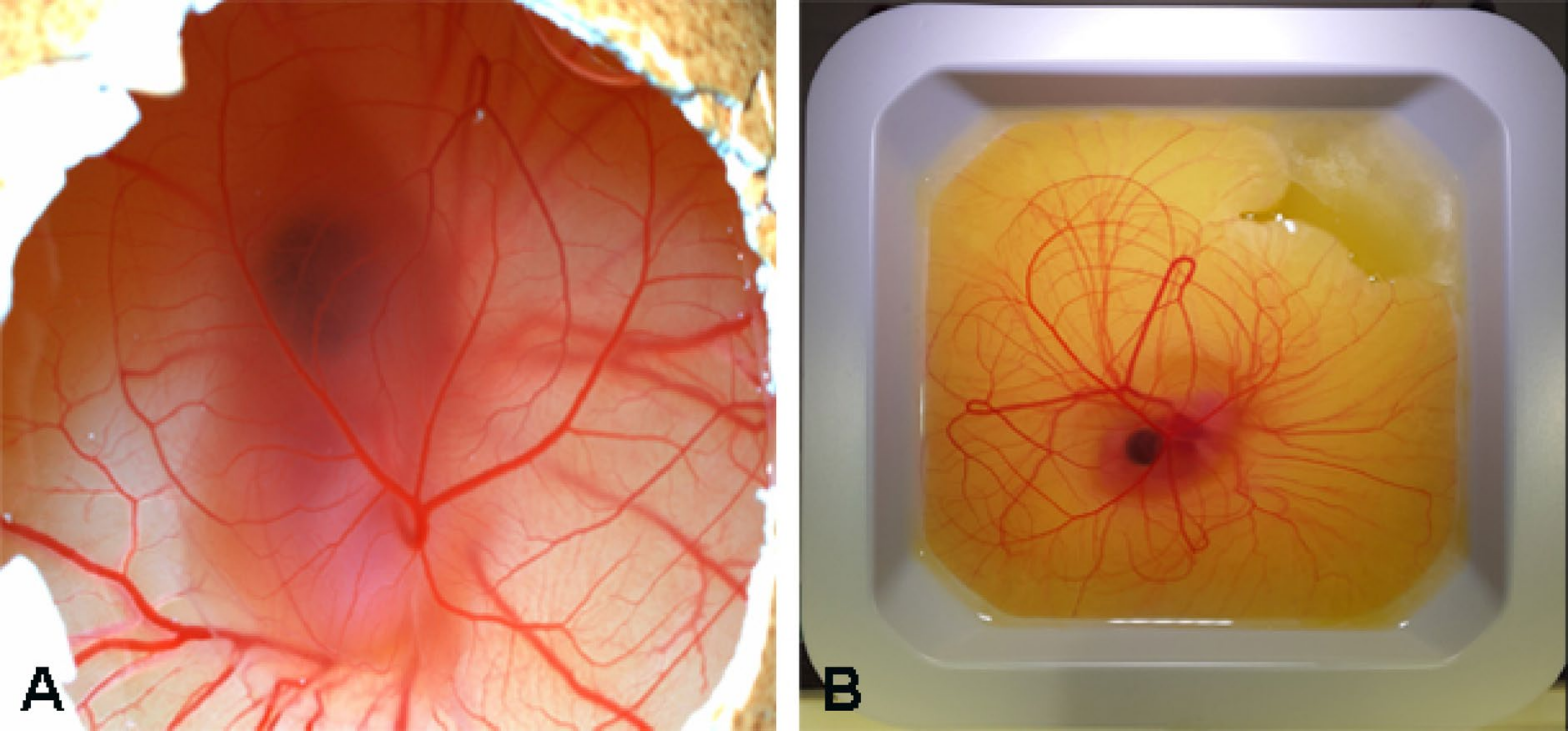
Imaging of the chicken chorioallantoic membrane (CAM) in in ovo (**A**) and ex ovo (**B**) conditions on ED6.

The results present a systematic comparison of CAM morphological and ultrastructural development under in ovo and ex ovo incubation conditions (Fig. 1) across defined embryonic stages.

### Blood vessel count

**Fig. 2:**
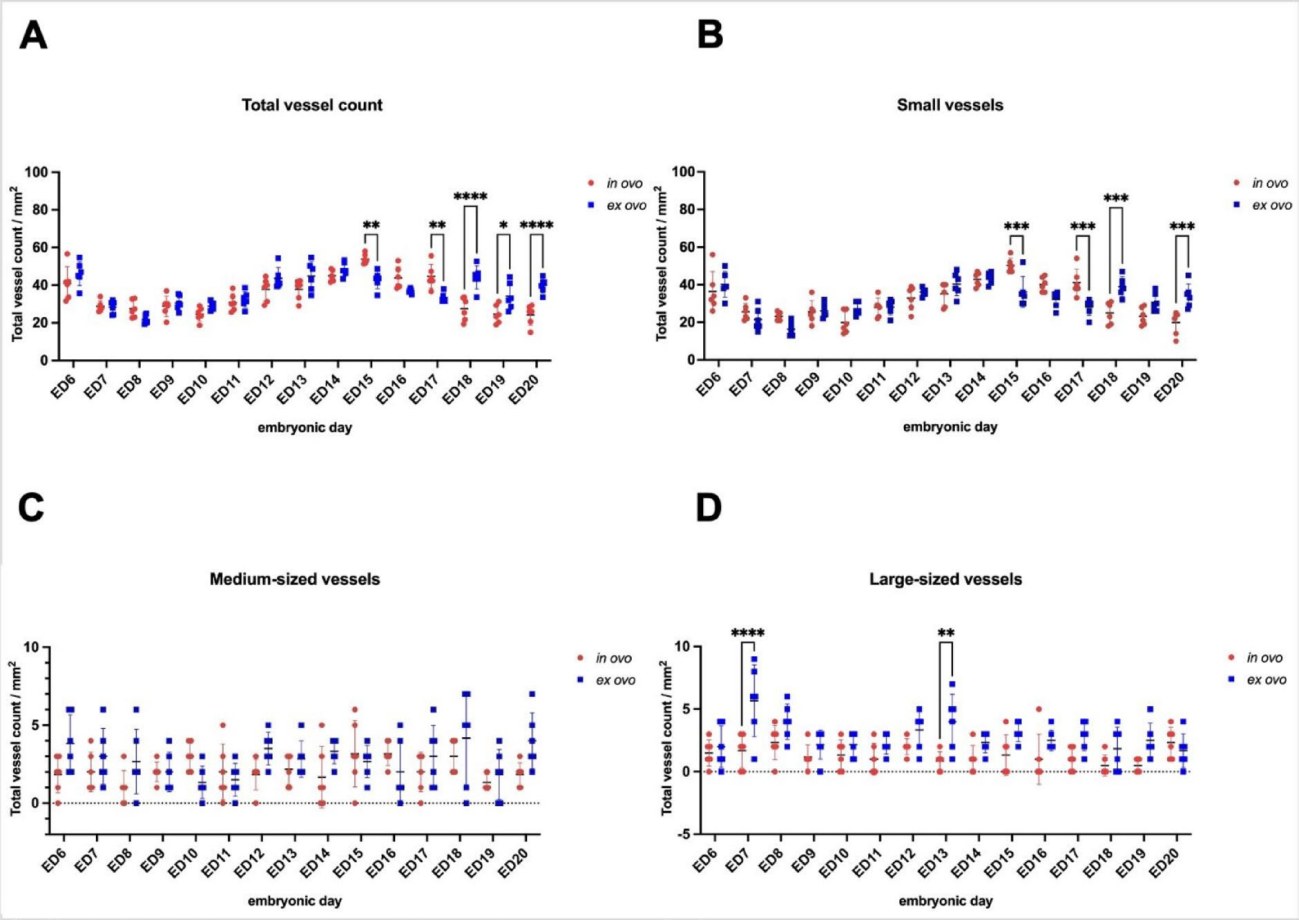
Developmental changes in CAM vascularization from ED6 to ED20. Total vessels counts (**A**) and size-stratified vessels counts (0–50 μm (**B**), 50–100 μm (**C**), and > 100 μm (**D**) were quantified from six microscopic fields per embryo, each with an area of 0.785 mm^2^, giving a total evaluated area of 4.71 mm^2^. For each embryo, counts from individual fields were averaged to obtain a single value prior to statistical analysis. Data are presented as mean ± SD (*n* = 6 embryos per group). Statistical analysis was performed using two-way ANOVA (factors: incubation condition and developmental day) followed by Sidak’s multiple comparisons test. Statistical significance is indicated as follows: *p < 0.05, **p < 0.01, ***p < 0.001, ****p < 0.0001.

Initial CAM formation was observed between ED4 and ED5, followed by an increase in total vessel count in both systems, which subsequently declined toward later developmental stages (Fig. 2A).

Pronounced divergence between the CAMs became evident after ED12. Between ED12 and ED15, vessels numbers in ex ovo CAMs remained relatively stable, whereas in ovo CAMs showed a more gradual increase, with a significant rise from ED10 onward, corresponding to the second phase of CAM development.

Peak vascularization in in ovo embryos occurred at ED15 (53.89 ± 2.60 vessels), whereas the maximum in ex ovo CAMs was observed at ED14 (48.39 ± 3.70 vessels). The lowest vessel counts were recorded at ED10 (24.56 ± 3.65) and ED20 (24.22 ± 5.42) in the in ovo system, and at ED8 (21.72 ± 2.38) in the ex ovo system.

Statistically significant differences between incubation systems were detected at ED15 and ED17-ED20 (*p* < 0.01–0.0001, Fig. 2A), consistent with the significant Time × Cultivation interaction revealed by two-way ANOVA (Table S1).

### Blood vessel diameter

Morphometric analysis of CAM vasculature across developmental stages revealed distinct blood vessel populations characterized by varying diameters and degrees of maturation. Based on lumen diameter, vessels were categorized into three groups: (1) small (0–50 μm), (2) medium (50–100 μm), and (3) large (> 100 μm). Across both in ovo and ex ovo incubation conditions, small vessels were the most abundant, followed by medium-sized vessels, while large vessels were the least frequent. This distribution pattern remained consistent irrespective of incubation conditions.

For small vessels (Fig. 2B), a significant developmental time effect was observed. Although no overall cultivation effect was detected. Statistically significant differences between in ovo and ex ovo CAMs occurred at ED15, ED17, ED18, and ED20 (*p* < 0.001), reflecting the significant interaction between time and incubation conditions (Table S1). For medium-sized vessels (Fig. 2C), no statistically significant time effect was observed. However, a significant cultivation effect was detected, indicating differences between incubation systems. Despite this, post hoc comparisons did not reveal consistent stage-specific differences across developmental days. In contrast, statistically significant differences were found in the distribution of large vessels between ex ovo CAMs at ED7 (*p* < 0.0001) and ED13 (*p* < 0.01; Fig. 2D). Additionally, both developmental time and cultivation significantly influenced large vessel distribution, with a significant interaction indicating divergent developmental dynamics between in ovo and ex ovo conditions.

### Total CAM thickness

**Fig. 3:**
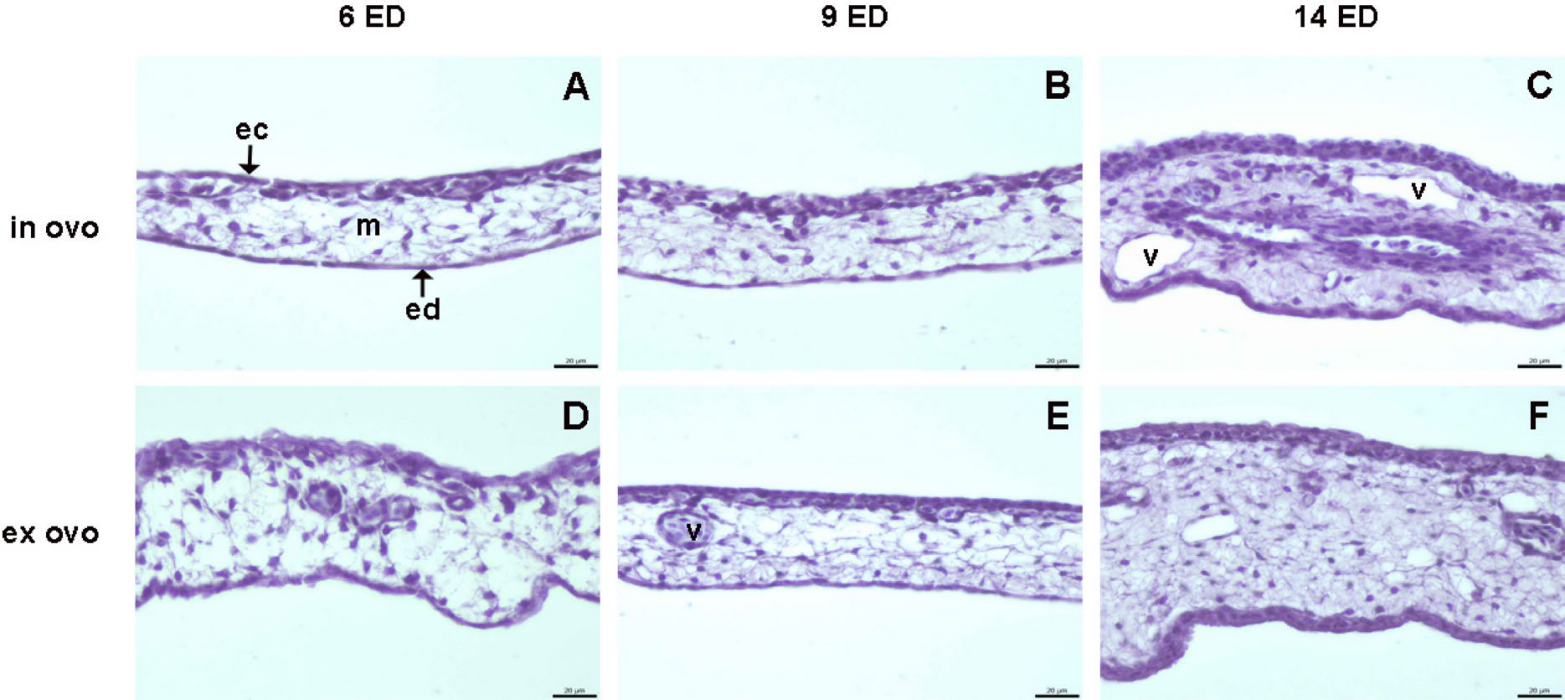
Histological organization of the CAM during embryonic development. Representative H&E-stained sections of the CAM at ED6 (**A**, **D**), ED9 (**B**, **E**) and ED14 (**C**, **F**) under in ovo (**A**–**C**) and ex ovo (**D**–**F**) conditions. The membrane exhibits the characteristic trilaminar structure consisting of ectoderm (ec), mesoderm (m), and endoderm (ed), with blood vessels (v) embedded within the mesodermal layer. Scale bar: 20 μm.

**Fig. 4:**
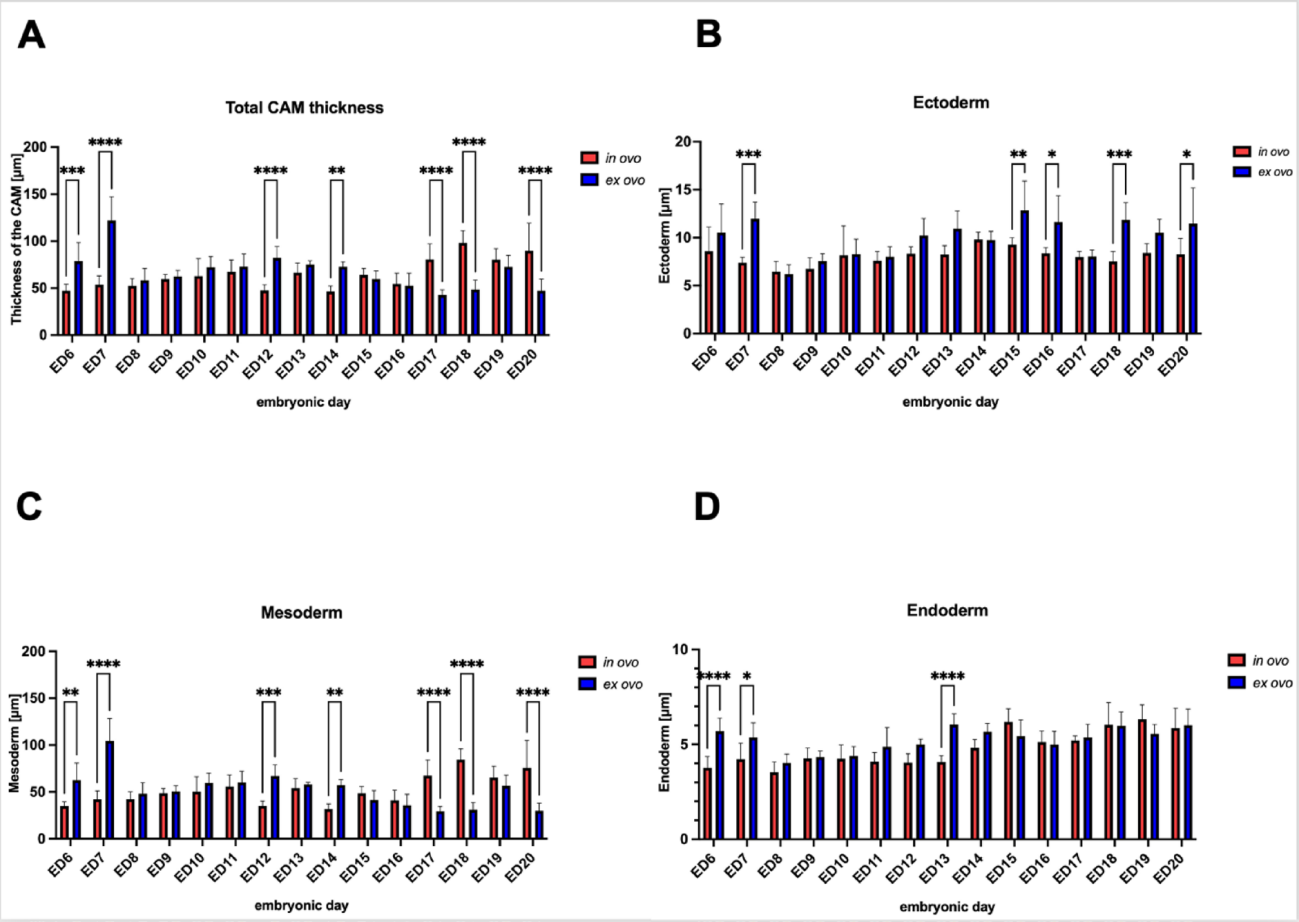
Quantitative assessment of CAM layer thickness during embryonic development. Total CAM thickness (**A**) and individual layer thicknesses—ectoderm (**B**), mesoderm (**C**), and endoderm (**D**)—were measured at indicated developmental stages. For each embryo (*n* = 6 peer group), six sections were evaluated, and five measurements per section were averaged to generate a single biological replicate. Data are presented as mean ± SD. Two-way ANOVA assessing the effects of incubation condition and developmental stage, including their interaction, was followed by Sidak’s multiple comparisons test. Statistical significance is indicated as follows: *p < 0.05, **p < 0.01, ***p < 0.001, ****p < 0.0001.

The overall thickness of the CAM varied substantially during embryonic development, following different temporal patterns under the two incubation conditions (Fig. 3). In in ovo embryos, CAM thickness ranged from 46.43 ± 5.80 μm at ED14 to a maximum of 98.07 ± 12.91 μm at ED18, showing a progressive increase toward later stages. In ex ovo embryos, thickness was highest at ED7 (121.89 ± 25.18 μm) and declined thereafter, reaching its lowest value at ED17 (42.78 ± 5.47 μm). Direct comparisons revealed significant differences (*p* < 0.0001) at ED7 and ED12, when ex ovo CAMs were thicker. Conversely, in ovo CAMs exceeded ex ovo thickness at ED17, ED18, and ED20 (Fig. 4A). These findings are consistent with the significant Time × Cultivation interaction detected by two-way ANOVA (Table S2).

### Ectodermal layer thickness

In in ovo embryos, ectodermal thickness increased gradually from 6.46 ± 1.06 μm at ED8 to a peak of 9.82 ± 0.75 μm at ED14, followed by moderate fluctuations. Under ex ovo conditions, thickness was similar at ED8 (6.21 ± 0.78 μm) but continued to rise, reaching a maximum of 12.86 ± 3.04 μm at ED15. Significant differences (*p* < 0.001) between the two systems were detected at ED7 and ED18. At later developmental stages (ED15, ED16, ED18, and ED20), the ectodermal layer was consistently thicker under ex ovo conditions (Fig. 4B).

### Mesodermal layer thickness

The mesoderm showed the highest variability among CAM layers (Fig. 4C). In ovo CAMs displayed progressive thickening, ranging from 31.79 ± 5.48 μm at ED14 to 84.51 ± 11.34 μm at ED18. In contrast, ex ovo CAMs exhibited earlier peaks, with the maximum value of 104.60 ± 23.73 μm recorded at ED7, followed by a decline toward later stages (minimum 29.37 ± 5.09 μm at ED17). Comparisons revealed significant difference (*p* < 0.0001) at ED7, with ex ovo CAMs being thicker. From ED17 to ED20, mesodermal thickness was significantly (*p* < 0.0001) greater in in ovo embryos.

### Endodermal layer thickness

In in ovo embryos, endodermal thickness increased steadily from 3.53 ± 0.55 μm at ED8 to a maximum of 6.33 ± 0.74 μm at ED19. Under ex ovo conditions, values ranged from 4.02 ± 0.48 μm at ED8 to 6.04 ± 0.57 μm at ED13, indicating an earlier peak compared with the in ovo system. Significant differences (*p* < 0.0001) were detected at ED6 and ED13, with thicker endoderm in the ex ovo group (Fig. 4D).

### Ultrastructural analysis of cellular and tissue architecture

**Fig. 5:**
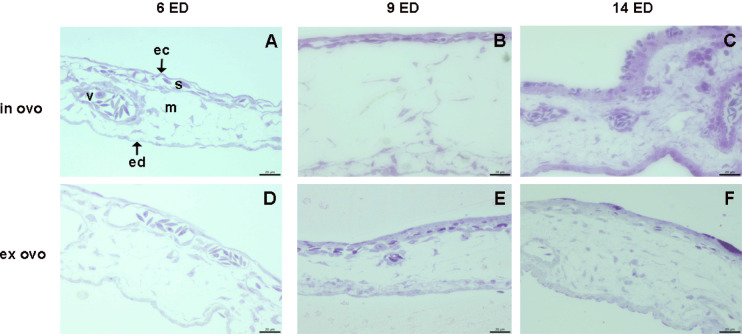
Semi-thin sections of the CAM samples selected for TEM at ED6 (**A**, **D**), ED9 (**B**, **E**) and ED14 (**C**, **F**) under in ovo and ex ovo incubation conditions. The CAM consists of three layers: ectoderm (ec), mesoderm (m), and endoderm (ed). Blood vessels (v) are visible within the mesodermal layer, and sinusoids (s) are presented in ectodermal layer. Scale bar: 20 μm.

Following histological assessment, transmission electron microscopy (TEM) was employed to further evaluate the ultrastructural organization of the CAM and to compare morphological differences under in ovo and ex ovo incubation conditions. TEM enabled high-resolution visualization of subcellular features associated with tissue differentiation, vascular organization, and epithelial–mesenchymal interactions at defined stages of CAM development. Embryonic days 6, 9, and 14 were selected to represent early differentiation, active angiogenesis, and advanced CAM maturation, respectively. Intermediate time points were omitted to minimize processing variability and to focus on biologically meaningful developmental transitions (Fig. 5).

**Fig. 6:**
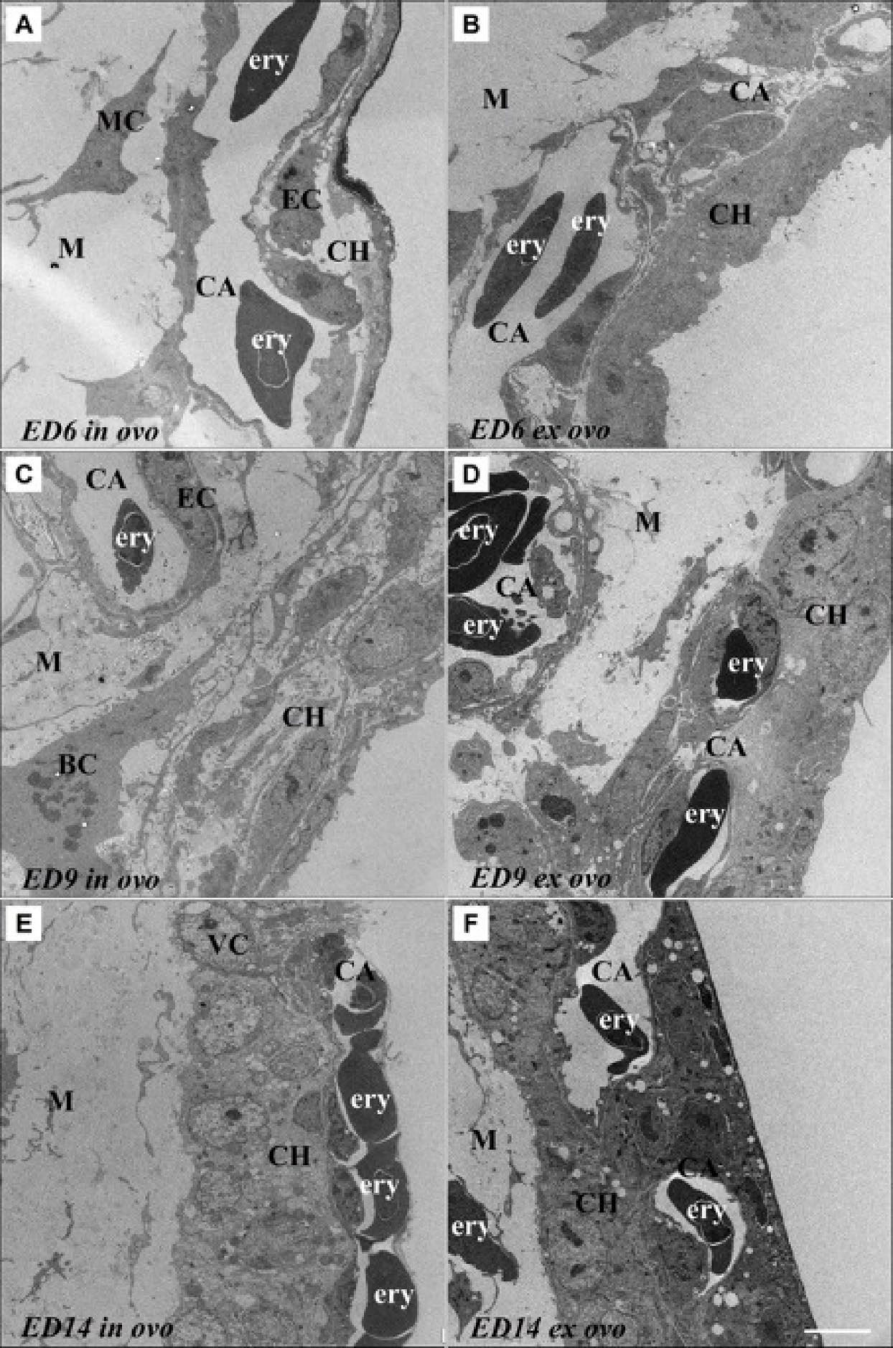
Electronograms of the CAM show changes in the ultrastructure of the chorionic epithelium depending on the day of embryonic development and incubation conditions. (**A**) ED6 in ovo, (**B**) ED6 ex ovo, (**C**) ED9 in ovo, (**D**) ED9 ex ovo, (**E**) ED14 in ovo, (**F**) ED14 ex ovo. CH: Chorion, M: Mesenchyme, CA: Capillaries, ery: erythrocytes, MC: Mesenchymal cells, EC: Endothelial cells, BC: Primary cells of the chorion, VC: Villous cavity cells. Scale bar: 5 μm.

The chorionic epithelium (CH; Fig. 6) in in ovo CAMs showed progressive thickening and differentiation, transitioning from a thin monolayer of flattened cells associated with developing capillaries (CA) to a multi-layered epithelium with highly differentiated cells and surface-oriented capillaries. In ex ovo CAMs, the chorion exhibited premature thickening, irregular stratification, and deeper vascular positioning, with a smoother epithelial surface; typical cell types such as villous cavity (VC) and basal cells (BC) were absent at later stages, indicating incomplete differentiation. The mesodermal layer (M) demonstrated incubation-dependent variability.

In ovo CAMs exhibited gradual thickening with well-organized vascular distribution—large blood vessels centrally and smaller capillaries subchorionically—and mesenchymal cells (MC) forming extensive cytoplasmic contacts, reaching maximal volume by ED9. Conversely, ex ovo CAMs showed accelerated mesodermal expansion, denser fibrous connective tissue, and centrally located vessels, reflecting faster but less coordinated angiogenesis.

**Fig. 7:**
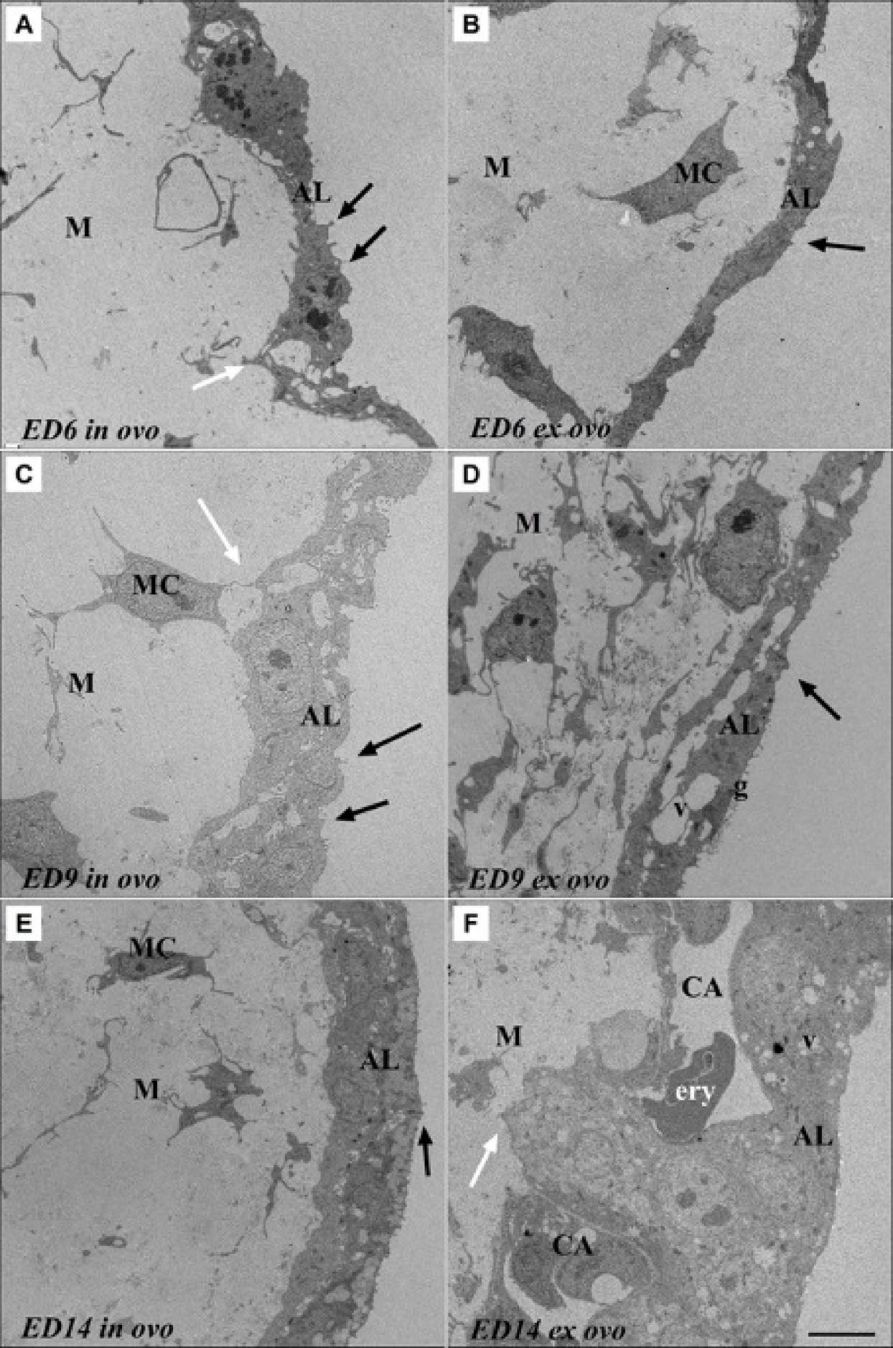
Electronograms of the CAM show changes in the ultrastructure of the allantoic epithelium depending on the day of embryonic development and incubation conditions. (**A**) ED6 in ovo, (**B**) ED6 ex ovo, (**C**) ED9 in ovo, (**D**) ED9 ex ovo, (**E**) ED14 in ovo, (**F**) ED14 ex ovo. M: Mesenchyme, AL: Allantois, CA: Capillaries, ery: erythrocytes, MC: Mesenchymal cells, EC: Endothelial cells, g: Glycogen-containing granules, v: Vacuoles, Filopodia (white ↓), Microvilli (black ↓). Scale bar: 5 μm.

The allantoic epithelium (AL; Fig. 7) developed from a single layer of fibroblast-like cells in both systems to a double-layered structure with glycogen (g) accumulation in in ovo CAMs, accompanied by a reduction of microvilli (black ↓) and filopodia (white ↓) as maturation progressed. In ex ovo CAMs, the allantois became irregularly multi-layered, with some cells extending into the mesenchyme and containing intratissue vessels, while microvilli and filopodia were reduced, reflecting impaired epithelial differentiation.

Overall, TEM analysis confirmed that the three-layered architecture of the CAM was maintained in both incubation conditions, while ex ovo cultivation accelerated mesodermal development but disrupted epithelial organization and vascular topology. These ultrastructural differences were consistent with histological observations of increased mesodermal thickness and altered vessel distribution under ex ovo conditions, providing a mechanistic basis for the observed disparities in tissue maturation, angiogenic patterning, and cellular differentiation between the two cultivation systems.

### Western blot analysis

**Fig. 8:**
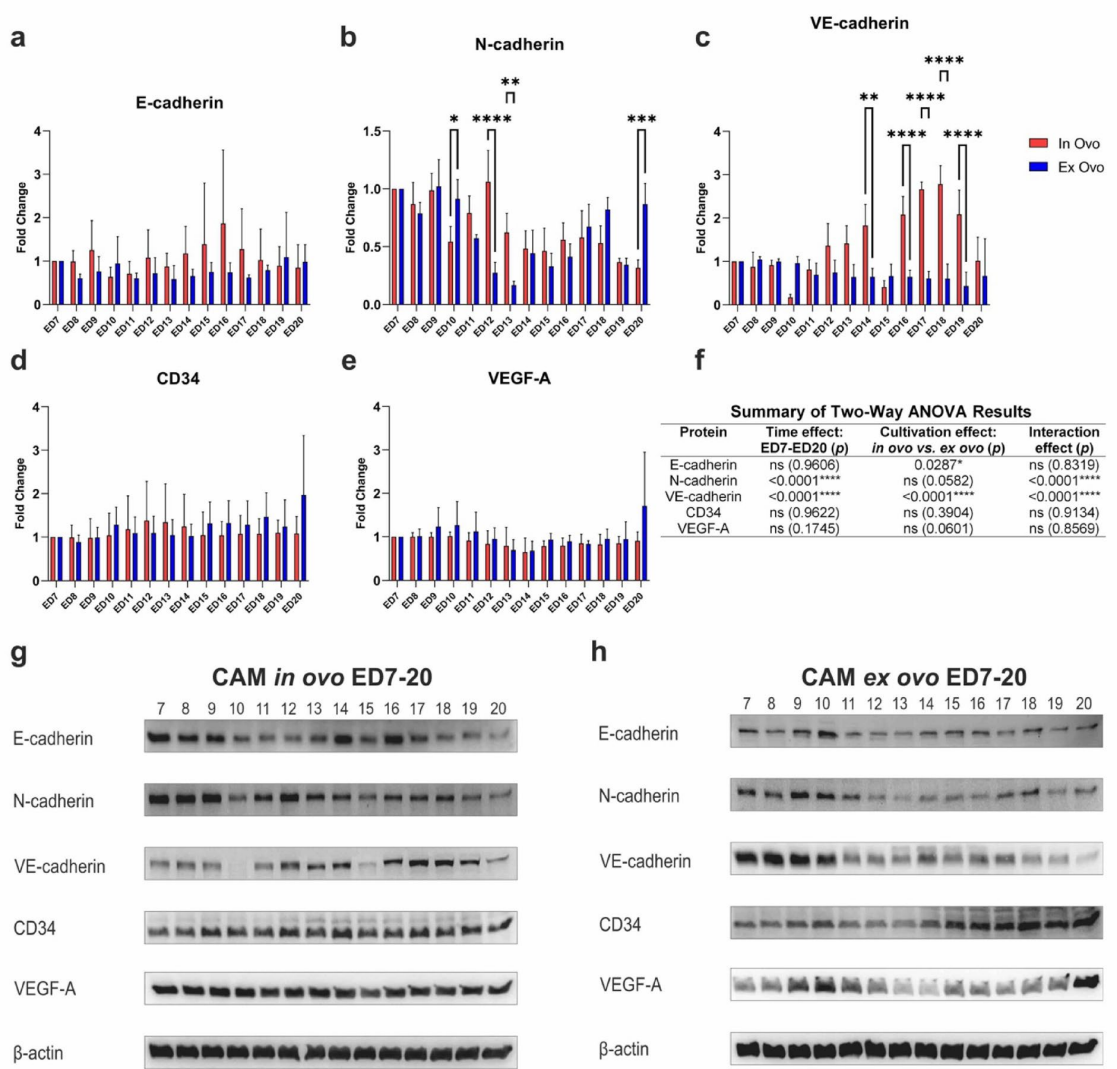
Western blot analysis of adhesion and angiogenesis-related proteins during CAM development under in ovo and ex ovo cultivation conditions. (**a**–**e**) Densitometric quantification of E-cadherin (**a**), N-Cadherin (**b**), VE-cadherin (**c**), CD34 (**d**), and VEGF-A (**e**) protein levels from ED7 to ED20. Protein expression was normalized to *β-*actin and is presented as fold change relative to ED7. Red bars represent in ovo cultivation and blue bars represent ex ovo cultivation. Data are shown as mean ± SD. Statistical significance between groups at individual time points is indicated by asterisks (*p < 0.05, **p < 0.01, ***p < 0.001, ****p < 0.0001). (**f**) Summary of two-way ANOVA evaluating the effects of developmental time (ED7-ED20), cultivation condition (in ovo vs. ex ovo), and their interaction on protein expression. (**g**) Representative Western blot image of CAM samples cultivated in ovo. (**h**) Representative Western blot images of CAM samples cultivated ex ovo. Cropped blots are shown; full-length, uncropped gels are provided in Figure S1.

Western blot analysis revealed protein-specific differences rather than general incubation-dependent differences in the expression patterns of adhesion molecules in the CAM (Fig. 8). Two-way ANOVA demonstrated a significant main effect of cultivation condition on E-cadherin expression (*p* < 0.001), with overall higher levels detected in in ovo embryos compared with ex ovo samples. In the in ovo group, a transient increase in E-cadherin levels was observed between ED14 and ED18. In contrast, E-cadherin expression remained relatively stable across embryonic development in the ex ovo group.

N-cadherin expression was significantly influenced by developmental stage (*p* < 0.0001), and a significant interaction between developmental stage and cultivation condition was detected (*p* < 0.0001), indicating distinct temporal expression dynamics in the two systems. Although no overall main effect of cultivation condition was observed (*p* > 0.05), post hoc comparisons revealed significant differences between in ovo and ex ovo embryos at specific embryonic days. Both conditions showed higher N-cadherin levels at earlier stages followed by a decline during mid-development, with recovery at later stages that differed between cultivation systems.

VE-cadherin expression was significantly affected by developmental stage (*p* < 0.0001), cultivation conditions (*p* < 0.0001), and their interaction (*p* < 0.0001). Overall, VE-cadherin levels were markedly higher in in ovo embryos. In ovo samples displayed a pronounced mid-to-late developmental increase, peaking between ED16 and ED19. Notably, VE-cadherin expression showed reproducible transient decreases around ED10 and ED15 before subsequent re-elevation. In contrast, ex ovo samples maintained consistently lower VE-cadherin levels compared to in ovo samples.

CD34 and VEGF-A expression was not significantly influenced by developmental stage, cultivation condition, or their interaction (*p* > 0.05 for all factors), indicating stable endothelial marker expression across both models.

### Comparison of CAM assay regarding mortality and tumor growth

**Fig. 9:**
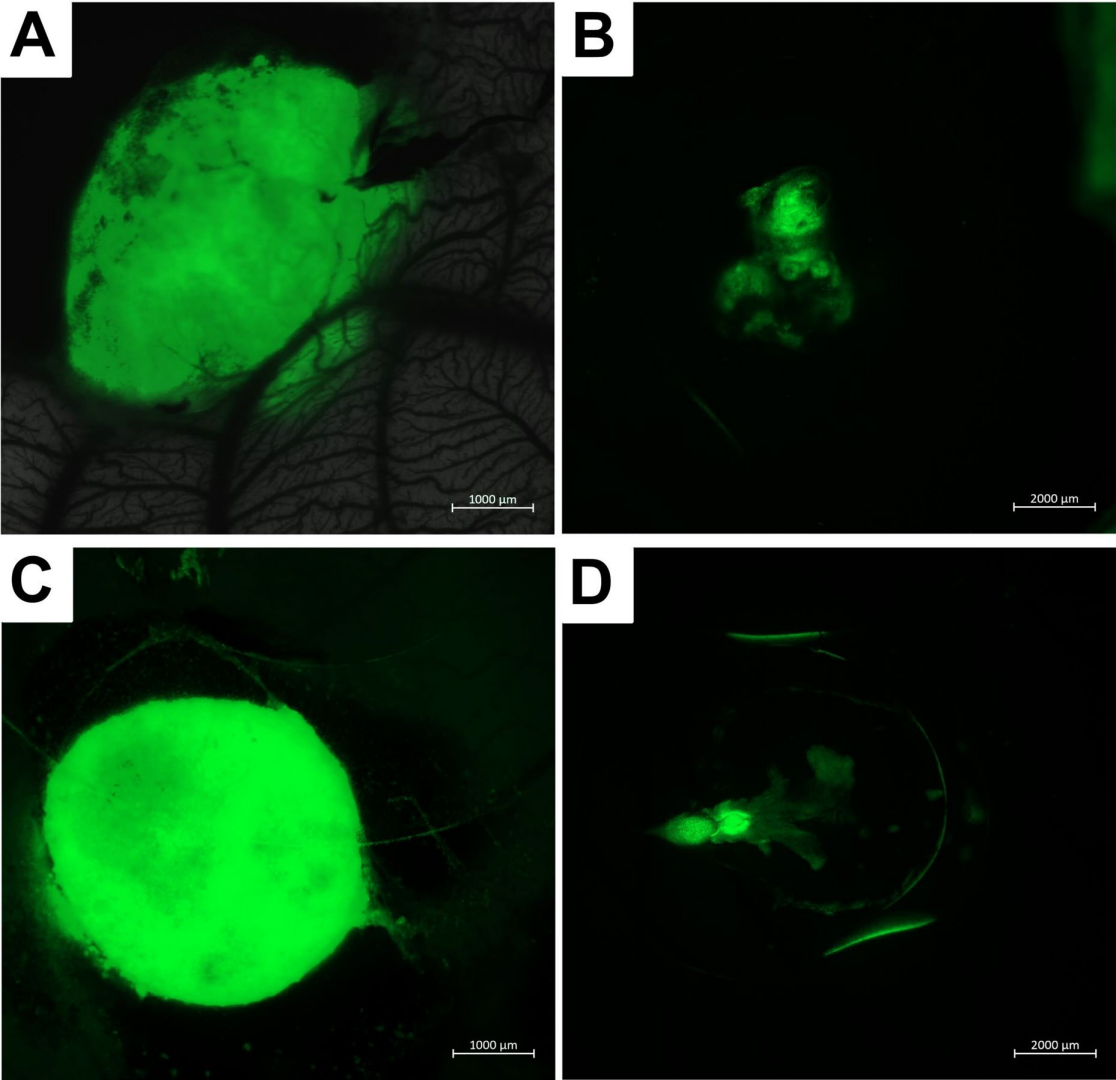
Fluorescence images of the GFP-labelled HT1080 human fibrosarcoma tumors in the in ovo CAM assay (**A**, **B**) and the ex ovo CAM assay (**C**, **D**). In both models, relatively larger tumors (**A**, **C**) and smaller tumors (**B**, **D**) were observed. Tumor sizes (mm^2^) were measured using ImageJ software. Scale bar: (**A**, **C**) 1000 μm; (**B**, **D**) 2000 μm.

**Fig. 10:**
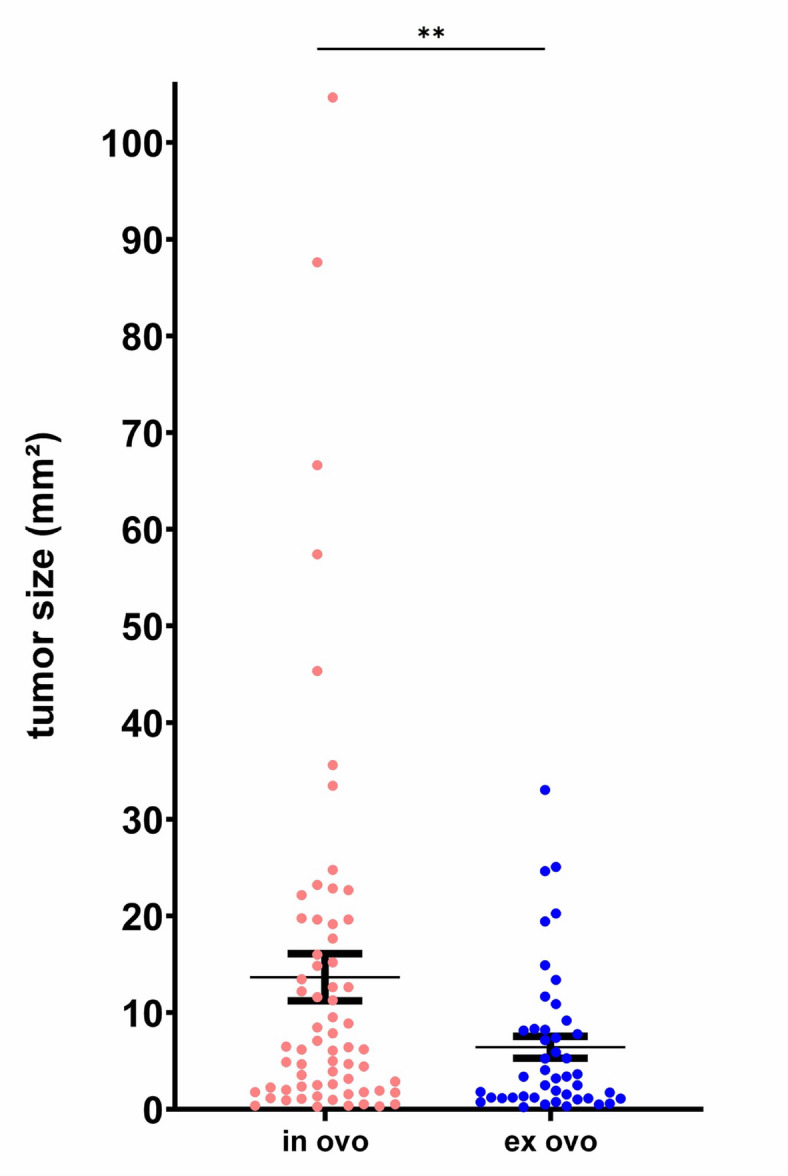
Tumor sizes in the in ovo (*n* = 66) and ex ovo (*n* = 51) CAM assay using HT1080 human fibrosarcoma cells. Individual data points represent raw tumor sizes (mm^2^). The horizontal lines represent the mean, and error bars indicate the standard error of the mean (SEM). The y-axis was segmented to accommodate outliers in the in ovo group. Statistical significance was determined using a linear mixed-effects model to Box-Cox-transformed data (**p < 0.01).

To compare the in ovo and the ex ovo CAM assay, the mortality rates, the tumor growth, and the success rate of tumor growth were investigated (Fig. 9). In the in ovo embryos, we observed a significantly higher survival rate, pre-inoculation (*p* < 0.0001), post-inoculation (*p* < 0.001), and overall (*p* < 0.0001) when compared to the ex ovo embryos (in ovo *n* =  150, ex ovo *n* = 182; Table 1). The in ovo embryos had a total mortality rate of 36.00%, a pre-inoculation mortality rate of 11.33% and a post-inoculation mortality rate of 27.81%. The ex ovo embryos had a total mortality rate of 58.79%, a pre-inoculation mortality rate of 26.37%, and a post-inoculation mortality rate of 44.03%. Tumors grew significantly larger in the in ovo compared to the ex ovo CAM model (13.65 mm^2^ vs. 6.42 mm^2^, *p* < 0.01; Fig. 10). This overall effect was validated across five independent experiments using a linear mixed-effects model to account for inter-batch variability (Figure S2). However, there was no significant difference between in ovo and ex ovo embryos in terms of the success rate of tumor growth (68.80% and 68.00%, respectively, *p* = 1).

**Table 1:** Survival, mortality and tumor growth rates in in ovo vs. ex ovo CAM assay using HT1080 human fibrosarcoma cells. Data are presented as observed events (n) relative to the surviving population (N) at each experimental step. Group comparisons were performed using Fisher’s exact test.

Parameter	In ovo			Ex ovo			*p*-value
	Count (*n*)	Total (*N*)	%	Count (*n*)	Total (*N*)	%	
Pre-inoculation							
Survival	133	150	88.70	134	182	73.60	*p* < 0.001
Mortality	17	150	11.33	48	182	26.37	–
Post-inoculation							
Survival	96	133	72.20	75	134	56.00	*p* = 0.007
Mortality	37	133	27.81	59	134	44.00	–
Overall experiment							
Survival	96	150	64.00	75	182	41.20	*p* < 0.0001
Mortality	54	150	36.00	107	182	58.79	–
Tumor evaluation							
Tumor growth observed	66	96	68.80	51	75	68.00	*p* = 1
No tumor growth observed	30	96	31.30	24	75	32.00	–

## Discussion

The present study demonstrates that the development of the CAM is markedly influenced by the incubation environment. To our knowledge, this study provides the first systematic, side-by-side comparison of in ovo and ex ovo CAM development under standardized conditions. Through morphometric, histological, ultrastructural, and protein expression analyses, we identified distinct developmental patterns and observed differences associated with the incubation environment. While in ovo incubation supported a more gradual development that peaked at later embryonic days, ex ovo conditions frequently led to earlier thickening of CAM layers, particularly the mesoderm and ectoderm, and altered vascular distribution.

Although the characteristic three-layer CAM structure was preserved in both models, the ex ovo incubation induced distinct morphological deviations^27–29^. Specifically, ex ovo CAMs exhibited irregular epithelial stratification, altered cell morphology, and reduced microvilli density in the ectoderm, particularly at later stages. In contrast, in ovo CAMs maintained a consistent epithelial architecture with well-preserved microvilli and tight cell junctions, features essential for maintaining barrier integrity and efficient gas exchange^30^. During incubation, embryonic gas exchange depends on the CAM, where diffusion across the eggshell is tightly coupled to cardiovascular convection. Ex ovo incubation removes this physiological interface, likely altering gas exchange dynamics and driving compensatory structural and vascular changes^31^. In ovo CAMs exhibited gradual mesodermal thickening that peaked later, consistent with the physiological timeline of vascular maturation^32^. In contrast, the mesoderm of ex ovo embryos showed increased thickness at earlier stages (ED6–ED14), accompanied by enhanced connective tissue deposition and centrally located blood vessels. This suggests that ex ovo incubation accelerates mesodermal development, potentially at the expense of normal vascular organization. The endoderm of ex ovo CAMs was initially thicker during early development; however, in ovo CAMs eventually surpassed ex ovo in thickness at later stages. This shift likely reflects the dynamic adaptation of the endoderm to metabolic demands and waste management, which becomes more critical as the embryo grows^33,34^. Because the ex ovo CAM is directly exposed to the external environment, early differences between incubation methods likely reflect increased evaporation and loss of the eggshell’s specialized role as a regulated diffusion interface for gas exchange^35–37^. These observations support a central role for the eggshell as a regulatory diffusion interface whose removal perturbs CAM tissue organization^37,38^.

Vascular development in the CAM can be understood as a highly dynamic process involving multiple sequential and overlapping steps: vessel formation, stabilization, branching, remodeling, pruning, and specialization^39,40^. During this process, vessels are specified into different calibres and types, including arteries, capillaries, and lymphatics, and recruit supporting cells such as smooth muscle cells and pericytes to ensure structural stability. These processes are particularly pronounced during early CAM development, where rapid vessel formation is followed by selective regression and remodeling, resulting in transient fluctuations in vessel numbers^30^. This framework helps explain the observed differences between in ovo and ex ovo CAMs, where ex ovo conditions accelerate mesodermal thickening and vascular organization, yet may disrupt normal vessel patterning, highlighting the importance of temporal coordination in angiogenesis for functional tissue maturation.

As the primary respiratory organ, the CAM forms early and exhibits diffusion-vascular coupling that is likely disrupted in ex ovo systems^41^. While the overall vessel count was higher in in ovo CAMs, the ex ovo model exhibited a more heterogeneous vascular distribution, indicating an accelerated angiogenic progression. Ultrastructural imaging further supported these findings, showing the earlier formation of a multi-layered chorion and altered capillary positioning in ex ovo CAMs, while in ovo CAMs retained the surface-oriented capillary arrangement, which maximizes gas exchange efficiency^42^.

Incubation-dependent differences extended to proteins regulating tissue organization. E-cadherin exhibited a biphasic trend in both systems, with an early increase during ED7–ED9 corresponding to active subepithelial proliferation, and a second increase between ED14–ED17 likely supporting enhanced epithelial cell–cell adhesion during continued CAM growth and structural organization^30^. Overall, changes were moderate, indicating largely preserved epithelial integrity in both models. In contrast, N-cadherin and VE-cadherin exhibited marked incubation-dependent differences, indicating altered endothelial stability and remodeling dynamics under ex ovo conditions. N-cadherin exhibited significant time-dependent variation with differing trajectories between in ovo and ex ovo CAMs, supporting the presence of distinct patterns of endothelial remodeling and cellular plasticity^43^. VE-cadherin showed the strongest developmental regulation. In in ovo CAMs, expression reproducibly decreased around ED10 and ED15, followed by subsequent re-elevation, consistent with transient adherens junction reorganization during vascular growth^44^. Lower levels toward late stages are in line with the known onset of physiological CAM regression before hatching^32^ and aligns with its dynamic regulation during early vessel morphogenesis^45^. Real-time PCR analysis of in ovo samples showed a similar trend (Figure S3), supporting that these changes reflect biological regulation rather than technical variability. CD34 and VEGF-A remained relatively stable, with no major differences between cultivation conditions, suggesting that the overall angiogenic program proceeds comparably in both systems. These incubation-dependent structural and molecular differences had clear functional consequences for tumor growth. We observed significantly higher tumor sizes in the in ovo model (13.65 mm^2^²) compared to the ex ovo model (6.42 mm^2^), despite identical inoculation conditions and timing. Tumor cells were grafted at ED9, a standard window corresponding to active CAM angiogenesis (ED4–ED11) and functional immunological immaturity (pre-ED15), allowing sufficient time for vascular connection and expansion^12,46,47^. Despite identical inoculation conditions, tumor growth differed markedly. Our data revealed that the CAM ectoderm is significantly thicker in ex ovo CAM assays during the critical tumor engraftment period (ED10–ED14) acting as a physical barrier, increasing the diffusion distance between the tumor and the underlying vascular mesoderm. We also observed this fact in our study after biomaterial implantation, where at least 120 h of incubation was required for DNA isolation from quail CAM tissue in ex ovo conditions^48^. In contrast, the thinner ectoderm of the in ovo model may facilitate rapid vascular recruitment. Notably, despite the differences in size, there was no difference in the tumor formation success rate between the two models.

Regarding mortality, the ex ovo CAM model exhibited a significantly higher overall mortality rate than the in ovo CAM model (58.79% vs. 36.00%). This difference is consistent with our pre-inoculation mortality (26.37% vs. 11.33%) and with previous reports of reduced survival in ex ovo systems compared with in ovo assays under normal conditions^9,10,21,22^. However, our overall mortality rates are not directly comparable to those in the literature due to inoculation of fibrosarcoma cells at ED9, increased mortality in ex ovo CAM assays—particularly at later developmental stages—is well documented and commonly attributed to insufficient calcium and phosphate uptake altered oxygen availability, limited space restricting embryo movement^22,35,49–51^. While alternative ex ovo setups using plastic cups have reported improved survival comparable to in ovo assays, the standard ex ovo method remains less efficient, requiring larger sample sizes to compensate for losses^16,51^. Consequently, the ex ovo model is less suitable for experiments involving rare or limited samples. Despite these drawbacks, it remains valuable for studies requiring easy visualization of the embryo and CAM as well as direct vascular access^49,52^.

This study has several limitations that should be considered when interpreting the results. First, functional validation was performed using a single tumor cell line, and although HT1080 cells represent a robust and angiogenesis-dependent xenograft model, responses may vary across tumor types or patient-derived samples. Second, molecular analyses were conducted on whole-CAM lysates, which do not allow cell-type-specific resolution of epithelial, endothelial, and mesenchymal contributions; however, this approach reflects the integrated tissue environment encountered by grafted cells and biomaterials. Moreover, a Western blot analysis confirmed differences in selected protein markers, additional validation using immunohistochemistry or immunofluorescence on CAM sections would provide valuable spatial information and should be considered in future studies. Third, ex ovo CAM culture systems with optimized temperature or humidity may partially mitigate mortality and developmental alterations. Finally, while this study focused on embryonic days relevant for angiogenesis and tumor growth, later embryonic development is not assessed due to the physiological regression of CAM vascular function and tissue organization. Future studies incorporating additional tumor models, cell-type-resolved analyses, and optimized ex ovo culture conditions will further refine understanding of incubation-dependent effects in CAM assays.

## Conclusions

In summary, our findings demonstrate that incubation conditions are a critical determinant of CAM morphology, vascular organization, and functional properties. The in ovo model supports gradual and well-coordinated maturation, characterized by organized vascular distribution, stable adhesion molecule expression, appropriate epithelial differentiation, higher embryo survival, and sustained tumor growth, providing a physiologically relevant and experimentally robust platform for long-term and translational studies. In contrast, the ex ovo model offers practical advantages, including improved visualization of the CAM, easier experimental manipulation, and direct vascular access. However, these benefits are accompanied by pronounced morphological and functional alterations, including thickening of the ectodermal layer and a lack of direct vessel-CAM contact. As a result, tissue integration and biocompatibility assessment of applied grafts or materials requires a longer experimental duration. Overall, in ovo and ex ovo CAM assays are not biologically equivalent, and model selection should be guided by the specific experimental objective rather than technical convenience. Collectively, these findings highlight the critical importance of selecting the appropriate CAM model according to specific experimental objectives and provide a mechanistic framework for understanding incubation-dependent differences in angiogenesis and wound healing, biocompatibility, and tumor biology.

## Methods

### In ovo CAM assay

Fertilized eggs from broiler chickens (Ross 308) were obtained from a local hatchery (Broeierij David nv, Tielt, Belgium) and transported under temperature-controlled conditions. After cleaning and disinfecting the eggshell with 10% povidone-iodine (Poviderm dermicum, 100 mg/mL Ecuphar, GmbH, Germany), the eggs were incubated in an upright position in a rotary incubator with forced air circulation and automatic tilting (once per hour), at 37.5 ± 0.5 °C and 70% relative humidity (RH). At ED3, the eggs were removed from the incubator and the eggshell at both the blunt and upper-facing side was disinfected using povidone-iodine. A small hole was drilled into the blunt end using a Dremel tool, and 2 mL of albumen was aspirated with sterile needle to reduce internal pressure and facilitate access to the developing embryo for subsequent interventions. Subsequently, a square window was made on the upward-facing side of the eggshell using a cut-off wheel. Both the window and the drilled hole were covered with laboratory tape, and the eggs were placed horizontally into the stationary incubator. Mortality rates were monitored regularly.

For evaluation of incubation-related effects on CAM development, embryos were not subjected to any in ovo manipulation.

### Ex ovo CAM assay

Eggs used for the ex ovo CAM incubation and assay were obtained from the same hatchery as those for the in ovo CAM experiment. After cleaning, the eggshell surface was disinfected using 10% povidone-iodine (Poviderm dermicum, 100 mg/mL Ecuphar, GmbH, Germany). The eggs were then incubated in a stationary incubator set at 37.5 ± 0.5 °C and 70% RH. At ED3, the eggs were removed from the incubator, and the downward-facing lateral side of the eggshell was disinfected with povidone-iodine. Subsequently, a small incision was made on the downwards-facing side of the eggshell using a cut-off wheel (Dremel). The egg contents, including the embryo, were carefully transferred to square weighing boats (86 × 86 × 25 mm, Z154881-500EA, Sigma-Aldrich, Darmstadt, Germany) with a corner cut-off. The sterile cover of a square Petri dish (SIMPD210-16, VWR International, Leuven, Belgium) was used as the lid. The embryos were then placed in sterile plastic boxes with drilled holes, and additional small containers filled with distilled water were added to the boxes to maintain high local humidity. The embryos were re-incubated under the same conditions (37.5 ± 0.5 °C and 70% RH). Mortality rates were monitored regularly throughout the process.

### Sample collection for histology and TEM analysis

In order to investigate morphological differences between the in ovo and the ex ovo CAM incubation conditions, 120 in ovo and 120 ex ovo embryos were used. On the day of collection (ED6–ED20), the eggs incubated under in ovo conditions were opened on the blunt end and then a biopsy of the CAM was performed. In the ex ovo group, the CAM was accessible. The excised CAM samples (*n* = 6 per ED) were fixed in 10% buffered formalin for histological analysis and in Karnovsky fixative (2% paraformaldehyde and 2.5% glutaraldehyde solution in 0.1 M Sodium Cacodylate buffer, pH 7.2–7.4; EMS, Hatfield, PA) for transmission electron microscopy analysis. The decapitation method was used to euthanize embryos.

### Histological and morphometric analysis

CAM tissue samples were collected from embryos between ED6 and ED20. Following fixation for 24 h in 10% buffered formalin, specimens were dehydrated in a graded ethanol series, cleared, and embedded in paraffin. Serial sections of 7 μm thickness were prepared using a rotary microtome (Leica RM2244, Leica Microsystems, Deer Park, IL, USA), deparaffinized, and rehydrated. Sections were stained with Mayer’s hemalum solution (Millipore Sigma, St. Louis, MO, USA) and Eosin (Sigma-Aldrich, St. Louis, MO, USA), dehydrated, and mounted with Entellan (Millipore Sigma, St. Louis, MO, USA). All slides were examined under a light microscope (Olympus CX43, Olympus, Tokyo, Japan) equipped with a digital camera (PROMICAM 3-5CP+, Promicra, Prague, Czech Republic) at 20× magnification.

Morphometric analysis was performed on six randomly selected specimens per embryonic day (*n* = 6). Hematoxylin-eosin-stained sections were evaluated in accordance with stereological principles. Vessel counts were obtained at 20× magnification in six fields of view, each with an area of 0.785 mm^2^, giving a total evaluated area of 4.71 mm^2^, with three repetitions per section. Subsequently, all detected vessels were measured in five directions and categorized into three groups: 0–50 μm, 50–100 μm, and > 100 μm. Thickness of the ectodermal, mesodermal, and endodermal layers was measured five times in six sections for each group. All measurements were independently performed by two researchers using the Olympus CX43 microscope and PROMICAM 3-5CP+ system with QuickPHOTO MICRO 3.2 software (Promicra, Prague, Czech Republic).

### Transmission electron microscopy

In order to evaluate the ultrastructural changes of the CAM during embryonic development under in ovo and ex ovo incubation conditions, the CAM tissue (maximum size 1 × 1 mm) was fixed with Karnovsky fixative at 4 °C for 12 h. Following this, the CAM samples were washed in 0.1 M sodium cacodylate buffer (4 × 20 min). Subsequently, the samples were post-fixed in 1% OsO_4_ (EMS, Hatfield, PA) for 2 h at room temperature (RT). The samples were then dehydrated in an ascending series of ethanol, supersaturated with acetone, and embedded in EPON (EMS, Hatfield, PA). The samples were incubated for three days at a temperature of 60 °C. Subsequently, semi-thin sections  (1500 nm; Fig. 5) and ultrathin sections  (90 nm) were prepared using Leica EM UC6 ultramicrotome (Leica Microsystems). The semithin sections were contrasted with toluidine blue at a temperature of 60 to 70° C for 3 min and evaluated with an Olympus BX61 light microscope (Olympus, Tokyo, Japan) with an Olympus DP73 digital camera (CellSens Dimension software). Ultrathin sections were contrasted for 45 s with uranyl acetate prepared in distilled water. The ultrastructure of the CAM tissue was assessed and documented with a JEM-1400Plus transmission electron microscope (JOEL Benelux, Belgium, Radius software).

### Protein lysate preparation and Western blot analysis

Western blotting was performed to evaluate the expression of proteins involved in epithelial-mesenchymal transition, angiogenesis, and cytoskeletal organization in CAM samples collected from ED7 to ED20. For each time point, three CAM samples were processed.

Protein lysates were prepared through a standardized protocol in order to ensure the integrity and reproducibility of the CAM samples. CAM tissues were excised from the surrounding membrane and transferred into 2 mL Eppendorf tubes filled with 1 mL of cold PBS, finely minced into fragments, and centrifuged at 4 °C (500 × g, 5 min). To eliminate residual erythrocytes, the pellets were treated with 1 mL of RBC lysing buffer (BioLegend, San Diego, CA, USA) for 5 min at RT. This process was followed by the addition of 500 µL of PBS, which halted the lysis process. The subsequent step involved centrifugation (500 × g, 5 min). In the event of residual red discoloration of the pellet being observed, an additional lysis step was performed. Pellets were dissolved in Laemmli sample buffer (100 mM TRIS-HCl, 10% glycerol, 2% SDS, pH 6.8) supplemented with protease and phosphatase inhibitors (Sigma Aldrich, Merck KGaA). Lysates were sonicated (QSonica, 40% amplitude, 30 s), incubated on ice (20 min), and purified by centrifugation (10,000 × g, 10 min, 4 °C). Supernatants were collected and stored at -20 °C until further analysis. The protein concentration was determined by the BCA assay (Pierce™ BCA Protein Assay Kit, Thermo Fisher Scientific, Waltham, MA, USA).

Equal amounts of protein (15 µg of total protein per sample) were resolved by SDS-PAGE on 10% Bis-Tris gel and transferred onto PVDF membranes using the iBlot2 dry blotting system (Thermo Fisher Scientific, Waltham, Massachusetts, USA). The PVDF membranes were rinsed with a solution of Tris-buffered saline containing 0.1% Tween-20 (TBS-T). Thereafter, they were blocked for 1 h at RT in TBS-T buffer supplemented with 5% non-fat dry milk or bovine serum albumin (NFDM/BSA) and subsequently incubated overnight at 4 °C with primary antibodies (Table 2). After three washes in TBS-T, membranes were incubated with horseradish peroxidase (HRP)-conjugated secondary antibodies for 1 h at RT. Protein bands were visualized using SuperSignal™ West Pico PLUS chemiluminescent substrate (Thermo Fisher Scientific) and imaged on an iBright FL1500 Imaging System (Thermo Fisher Scientific). The β-actin was used as a loading control.

**Table 2:** Antibodies used for Western blot analysis.

Primary antibody	Host	Isotype	Dilution	Cat. No.	Clonality	Produced by
E-Cadherin	Rabbit	IgG	1:1000	3195	Monoclonal	CST, Danvers, MA, USA
N-Cadherin	Rabbit	IgG	1:1000	13,116	Monoclonal	CST, Danvers, MA, USA
VE-Cadherin	Rabbit	IgG	1:1000	ab33168	Polyclonal	Abcam, Cambridge, UK
CD34	Mouse	IgG	1:1000	MCA5936GA	Monoclonal	Bio-Rad, Hercules, CA, USA
VEGF-A	Rabbit	IgG	1:1000	ab214424	Monoclonal	Abcam, Cambridge, UK
β-actin	Rabbit	IgG	1:1000	8457	Monoclonal	CST, Danvers, MA, USA

Secondary antibody	Host	Isotype	Dilution	Cat. No.	Produced by
Anti-rabbit IgG, HRP-linked	Goat	IgG	1:1000	7074	CST, Danvers, MA, USA
Anti-mouse IgG, HRP-linked	Horse	IgG	1:1000	7076	CST, Danvers, MA, USA

### Cell culture

GFP-labelled human fibrosarcoma HT-1080 cells (ATCC, Rockville, MD) were cultured in RPMI medium (Gibco™, Waltham, MA, USA) supplemented with 1% penicillin/streptomycin (10,000 U/mL, Gibco™, Waltham, MA, USA), 1% HEPES (1 M, Gibco™, Waltham, MA, USA), 1% sodium pyruvate (100 mM, Gibco™, Waltham, MA, USA), 1% Minimum Essential Medium (MEM) Non-Essential Amino Acids Solution (100x, Gibco™, Waltham, MA, USA) and 10% fetal bovine serum (FBS, Biowest, Nuaillé, France). Cell cultures were maintained at 37 °C with 5% CO_2_ and passaged when reaching 80–90% confluency.

### Inoculation of HT1080 cells

To investigate functional differences between the in ovo and the ex ovo CAM assays, HT1080 cells were placed on the CAM of 182 ex ovo and 150 in ovo embryos. On ED9, cells were trypsinized with trypsin-EDTA 0.25% containing phenol red (Gibco™, Waltham, MA, USA) and resuspended in growth medium. Cell concentration and viability were determined using a Burker chamber in combination with trypan blue. For each embryo 5 × 10^5^ in 25 µL of growth medium was prepared. All cell manipulations were performed aseptically under a laminar flow hood.

Embryos were removed from the stationary incubator in groups of four and placed under the laminar flow hood. The lid of the weigh boat was removed to expose the embryo, and a small abrasion was made on the CAM between two major blood vessels using a cotton swab. A sterilized silicone ring (internal diameter of 7 mm, external diameter of 9 mm, thickness 100 μm) was placed over this abrasion, and the prepared cells were pipetted into the center of this ring. After this procedure, the embryos were returned to the stationary incubator and monitored regularly for mortality. On ED15, imaging was performed, and embryos were euthanized by decapitation.

### Imaging of the tumors

Images were taken using an Axio Zoom V16 fluorescent stereomicroscope (Leica Microsystems GmbH, Wetzlar, Germany) to detect GFP-labelled HT1080 cells and tumors formed by these cancer cells. These images were then analyzed using the Fiji is Just ImageJ software (version 1.54f, National Institute of Health, USA) by calculating the surface in mm^2^^53^.

**Fig. 11:**
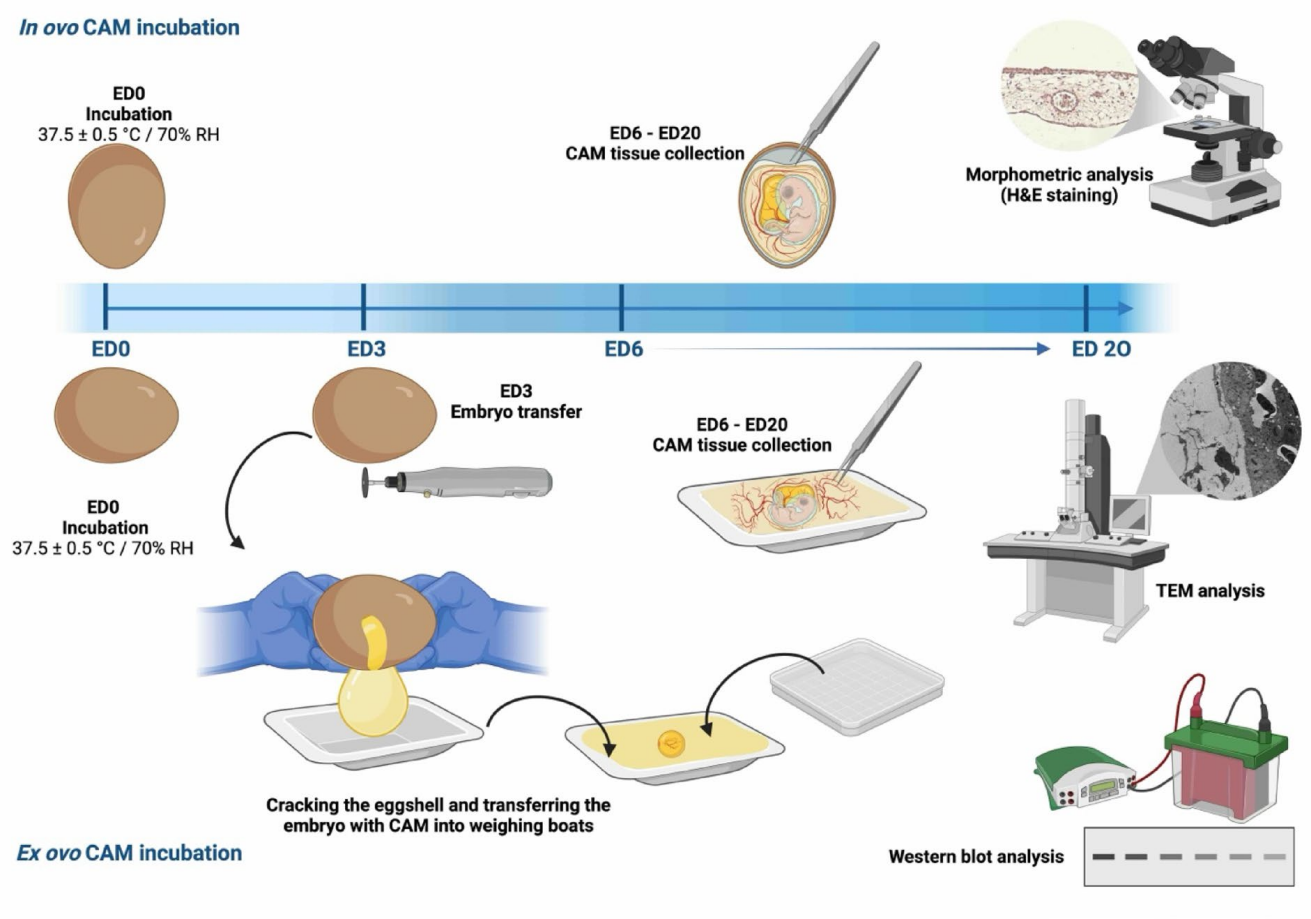
Overview of the experimental workflow for the comparative analysis of in ovo and ex ovo CAM development, integrating morphometric, histological, ultrastructural, and molecular analyses to assess incubation-dependent differences (created with BioRender.com).

**Fig. 12:**
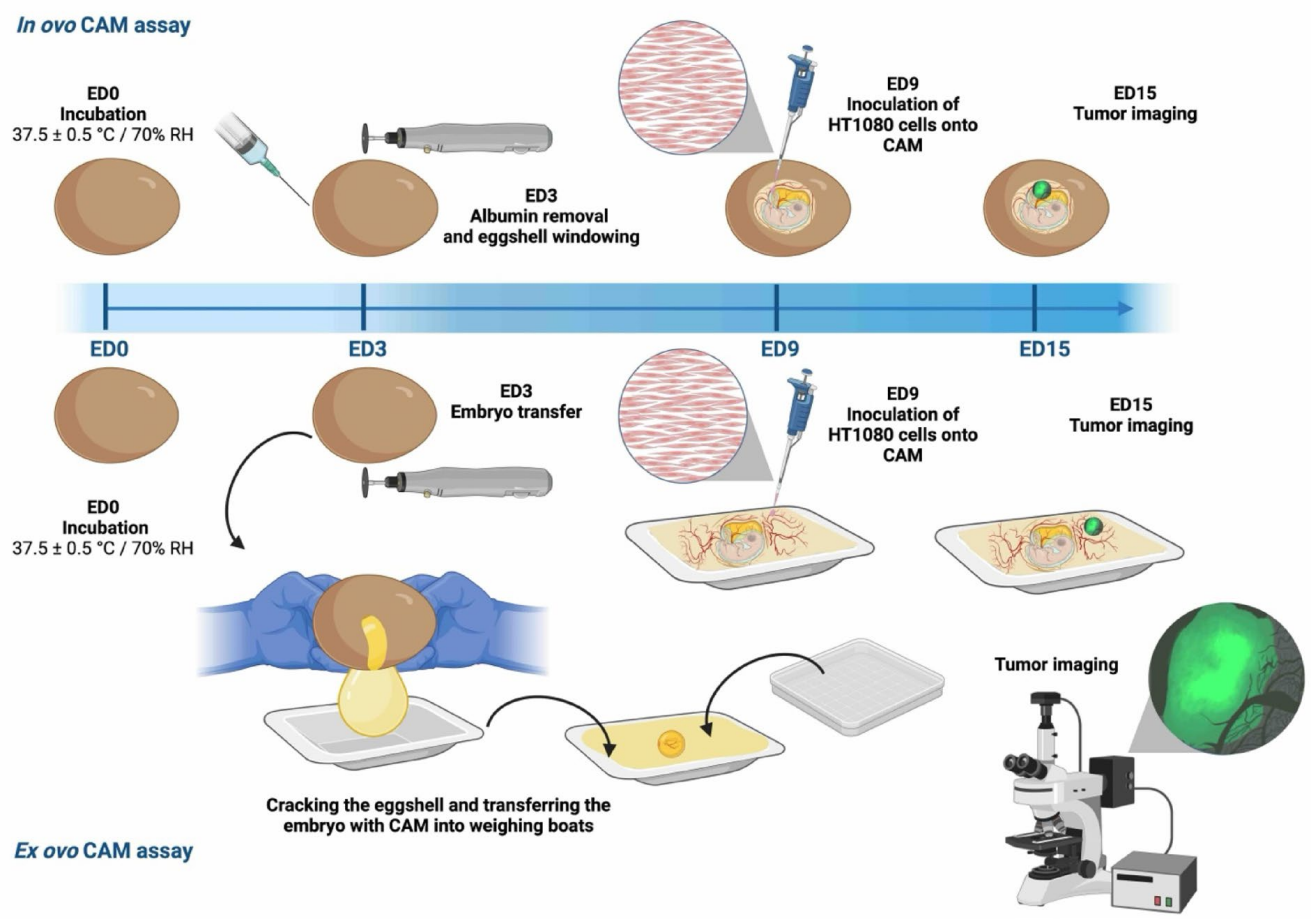
Experimental workflow for investigating functional differences between in ovo and ex ovo CAM assays, including tumor xenograft experiments to evaluate the impact of incubation conditions on angiogenesis and tumor growth (created with BioRender.com).

An overview of the experimental design and workflow employed in this study is schematically presented in Figs. 11 and 12.

### Statistical analysis

Statistical analyses of morphological features between in ovo and ex ovo CAM development were performed using GraphPad Prism 11 (GraphPad Software, LLC, San Diego, CA, USA). Data are presented as mean ± standard deviation (SD) from three independent experiments. Normality of the data was assessed with the Shapiro-Wilk test. Differences among groups were assessed using two-way ANOVA with developmental time and incubation conditions (in ovo vs. ex ovo) as factors, followed by Sidak’s multiple comparisons test as a post hoc analysis. Densitometric data obtained from Western blot analyses were evaluated using two-way ANOVA with developmental time and incubation conditions (in ovo vs. ex ovo) as factors, followed by Bonferroni’s multiple comparisons test. Data for the functional comparison between both in ovo and ex ovo CAM assays was obtained from five independent experiments, with eggs from both models originating from the same batch in each experiment. Normality of the data was assessed with Shapiro-Wilk test. As several datasets deviated from a Gaussian distribution, non-parametric tests were applied. Differences among multiple groups were assessed using the Kruskal-Wallis test. Tumor size data were Box-Cox transformed and analyzed using a linear mixed-effects model. Contingency tables used to evaluate mortality and tumor growth success rates, with Fisher’s exact test applied to assess statistical significance. The threshold for statistical significance was set at *p < 0.05, **p < 0.01, ***p < 0.001, ****p < 0.0001.

## Electronic Supplementary Material

Below is the link to the electronic supplementary material.

Supplementary Material 1

Supplementary Material 2

Supplementary Material 3

Supplementary Material 4

Supplementary Material 5

## Data Availability

The datasets generated and/or analyzed during the current study are included in the article and its Supplementary Materials. Further data are available from the corresponding authors upon reasonable request.
