# Supplementary material for: In ovo versus ex ovo incubation differentially shapes chorioallantoic membrane maturation, angiogenesis, and tumor growth
Source: Sci Rep. 2026 Apr 25;16:19221. doi: 10.1038/s41598-026-49692-9 (PMC13284325; doi:10.1038/s41598-026-49692-9)
Supplement: Supplementary file 1 — Supplementary Material 1 [file 41598_2026_49692_MOESM1_ESM.pdf]

Supplementary material: *Demcisakova et al. In ovo versus ex ovo incubation differentially shapes chorioallantoic membrane maturation, angiogenesis, and tumor growth*

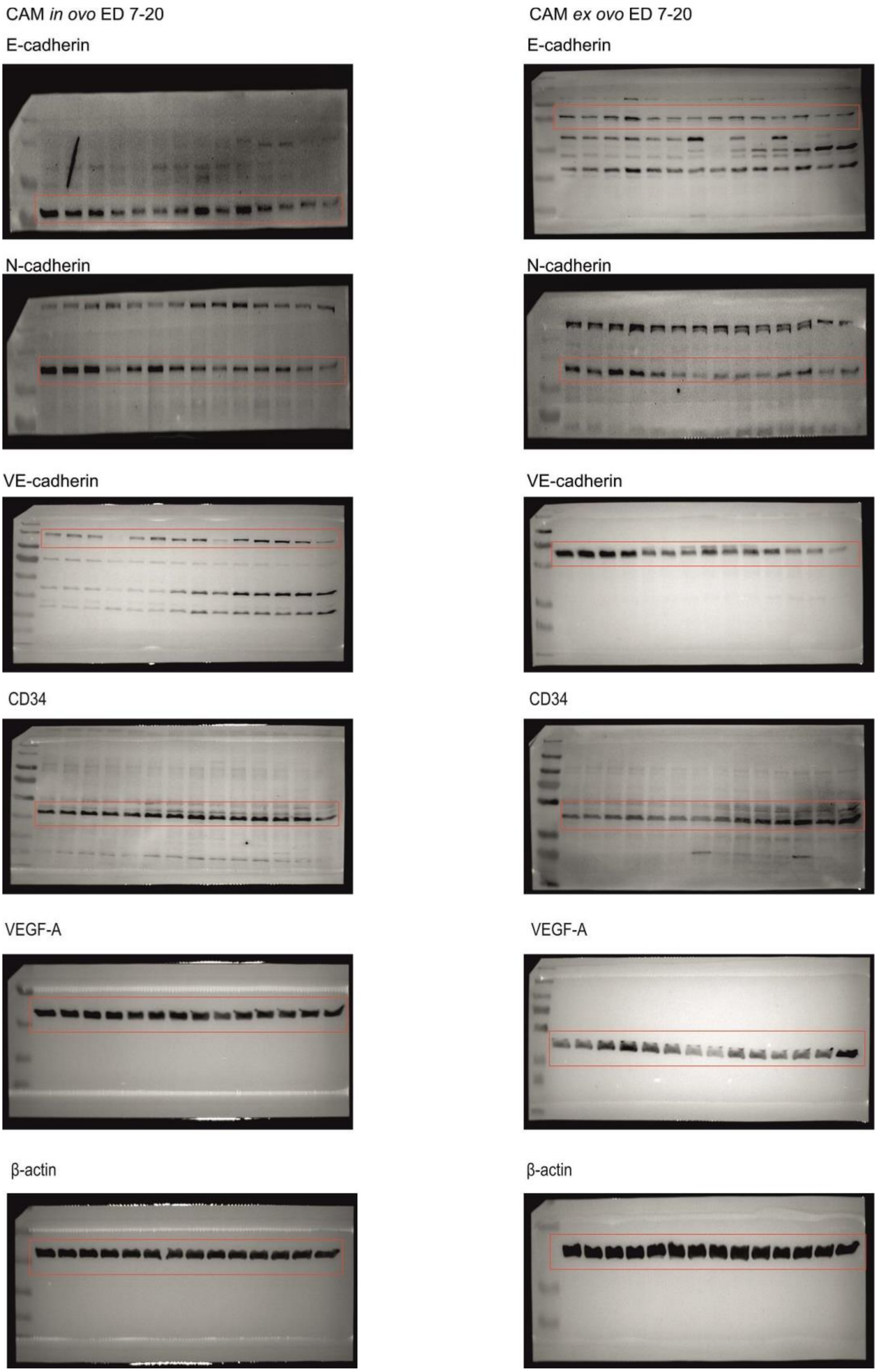

Figure S1. Full-length (uncropped) Western blot images of the analyzed proteins in CAM samples obtained under *in ovo* and *ex ovo* incubation conditions.
