# Supplementary material for: In ovo versus ex ovo incubation differentially shapes chorioallantoic membrane maturation, angiogenesis, and tumor growth
Source: Sci Rep. 2026 Apr 25;16:19221. doi: 10.1038/s41598-026-49692-9 (PMC13284325; doi:10.1038/s41598-026-49692-9)
Supplement: Supplementary file 2 — Supplementary Material 2 [file 41598_2026_49692_MOESM2_ESM.pdf]

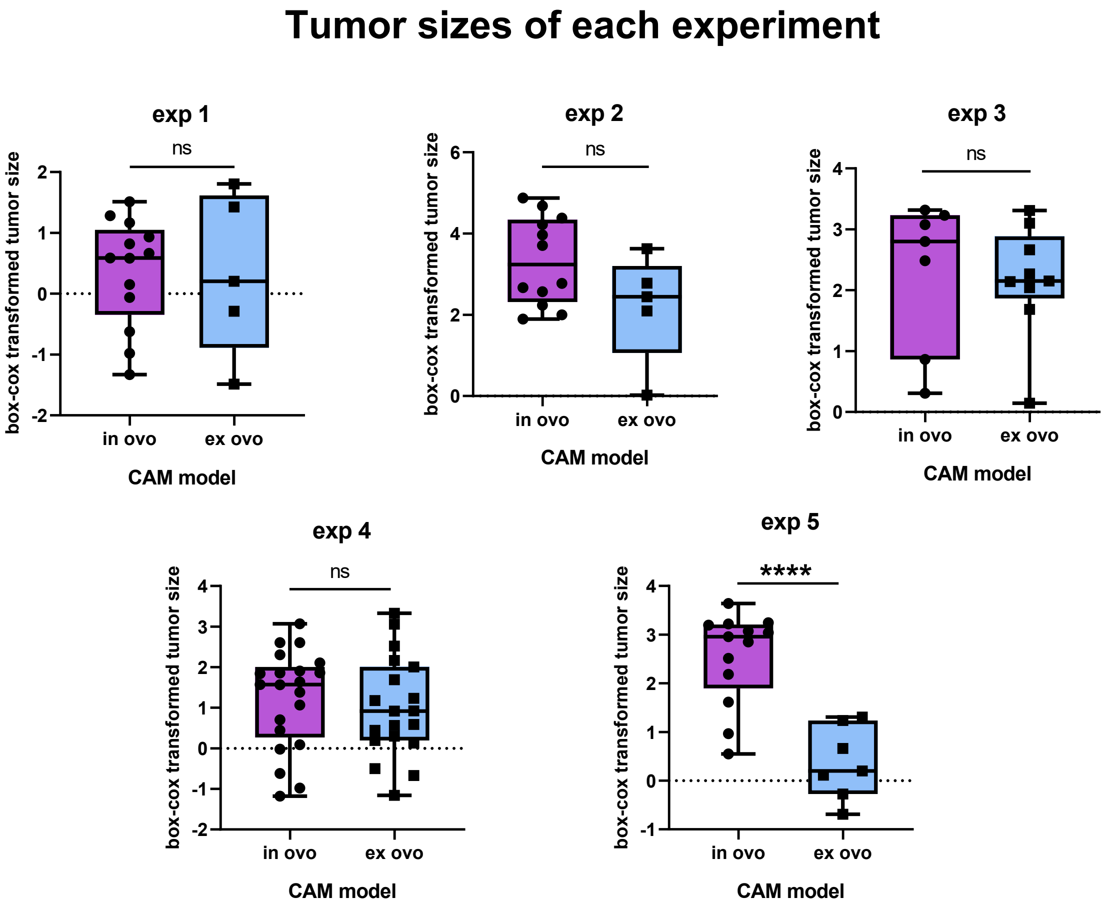

Figure S2. Tumor sizes across individual independent experiments. Box-Cox transformed tumor sizes comparing the *in ovo* and *ex ovo* CAM models across five separate experimental batches. Boxplots display the median and interquartile range, with points representing individual tumors. Statistical comparisons within each experiment were performed using unpaired t-tests (ns = not significant; ****$p < 0.001$).
