# Supplementary material for: In ovo versus ex ovo incubation differentially shapes chorioallantoic membrane maturation, angiogenesis, and tumor growth
Source: Sci Rep. 2026 Apr 25;16:19221. doi: 10.1038/s41598-026-49692-9 (PMC13284325; doi:10.1038/s41598-026-49692-9)
Supplement: Supplementary file 3 — Supplementary Material 3 [file 41598_2026_49692_MOESM3_ESM.pdf]

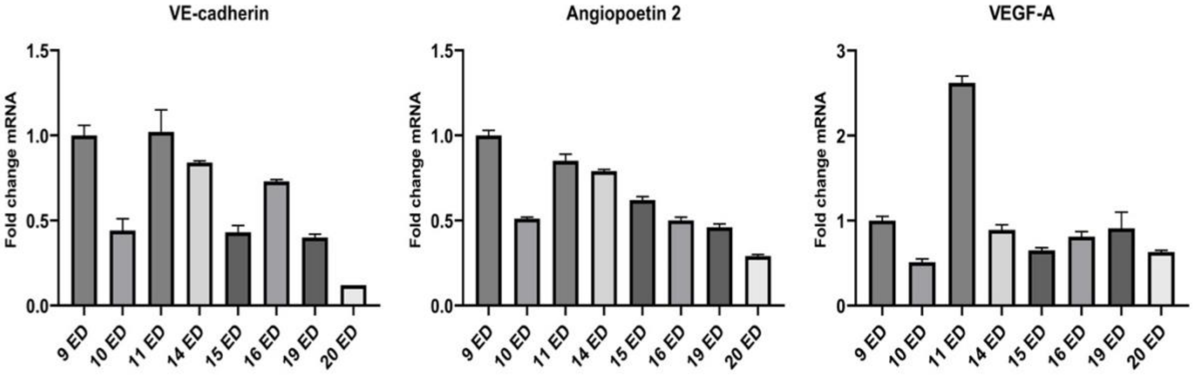

Figure S3. Real-time PCR analysis of gene expression in CAM (*in ovo*). Relative mRNA expression of VE-cadherin, Angiopoietin-2, and VEGF-A in the CAM during embryonic development (ED9–ED20) under *in ovo* conditions, determined by real-time PCR. Data are shown as mean ± SD.
