# Supplementary material for: In ovo versus ex ovo incubation differentially shapes chorioallantoic membrane maturation, angiogenesis, and tumor growth
Source: Sci Rep. 2026 Apr 25;16:19221. doi: 10.1038/s41598-026-49692-9 (PMC13284325; doi:10.1038/s41598-026-49692-9)
Supplement: Supplementary file 4 — Supplementary Material 4 [file 41598_2026_49692_MOESM4_ESM.pdf]

Supplementary material: *Demcisakova et al. In ovo versus ex ovo incubation differentially shapes chorioallantoic membrane maturation, angiogenesis, and tumor growth*

**Vascular parameters**	**Time effect: ED6-ED20 (*p*)**	**Cultivation effect: *in ovo* vs. *ex ovo* (*p*)**	**Interaction effect (*p*)**
Number of blood vessels	<0.0001****	<0.01**	<0.0001****
Small vessels	<0.0001****	ns (0.3738)	<0.0001****
Medium-sized vessels	ns (0.1070)	<0.01**	<0.05*
Large vessels	<0.001***	<0.0001****	<0.05*

Table 1. Two-way ANOVA analysis of CAM vascular parameters evaluating the effects of developmental time (ED6-ED20), incubation system (*in ovo* vs. *ex ovo*), and their interaction (Time x Cultivation). P-values correspond to main effects and the interaction term.
