# Supplementary material for: In ovo versus ex ovo incubation differentially shapes chorioallantoic membrane maturation, angiogenesis, and tumor growth
Source: Sci Rep. 2026 Apr 25;16:19221. doi: 10.1038/s41598-026-49692-9 (PMC13284325; doi:10.1038/s41598-026-49692-9)
Supplement: Supplementary file 5 — Supplementary Material 5 [file 41598_2026_49692_MOESM5_ESM.pdf]

Supplementary material: *Demcisakova et al. In ovo versus ex ovo incubation differentially shapes chorioallantoic membrane maturation, angiogenesis, and tumor growth*

**Structural parameters**	**Time effect: ED6-ED20 (*p*)**	**Cultivation effect: *in ovo* vs. *ex ovo* (*p*)**	**Interaction effect (*p*)**
CAM Thickness	<0.0001****	ns (0.0853)	<0.0001****
Ectoderm	<0.0001****	<0.0001****	<0.001***
Mesoderm	<0.0001****	ns (0.6029)	<0.0001****
Endoderm	<0.0001****	<0.0001****	<0.0001****

Table S2. Two-way ANOVA analysis of CAM layer thickness evaluating the effects of developmental time (ED6-ED20), incubation system (*in ovo* vs. *ex ovo*), and their interaction (Time x Cultivation). P-values correspond to main effects and the interaction term.
